# High Infection Rates for Adult Macaques after Intravaginal or Intrarectal Inoculation with Zika Virus

**DOI:** 10.3201/eid2308.170036

**Published:** 2017-08

**Authors:** Andrew D. Haddow, Aysegul Nalca, Franco D. Rossi, Lynn J. Miller, Michael R. Wiley, Unai Perez-Sautu, Samuel C. Washington, Sarah L. Norris, Suzanne E. Wollen-Roberts, Joshua D. Shamblin, Adrienne E. Kimmel, Holly A. Bloomfield, Stephanie M. Valdez, Thomas R. Sprague, Lucia M. Principe, Stephanie A. Bellanca, Stephanie S. Cinkovich, Luis Lugo-Roman, Lisa H. Cazares, William D. Pratt, Gustavo F. Palacios, Sina Bavari, M. Louise Pitt, Farooq Nasar

**Affiliations:** United States Army Medical Research Institute of Infectious Diseases, Frederick, Maryland, USA

**Keywords:** Zika virus, viruses, arbovirus, infection rates, macaques, rhesus macaques, cynomolgus macaques, nonhuman primates, intravaginal inoculation, intrarectal inoculation, sexual transmission, mosquitoes, vector-borne infections, zoonoses

## Abstract

Unprotected sexual intercourse between persons residing in or traveling from regions with Zika virus transmission is a risk factor for infection. To model risk for infection after sexual intercourse, we inoculated rhesus and cynomolgus macaques with Zika virus by intravaginal or intrarectal routes. In macaques inoculated intravaginally, we detected viremia and virus RNA in 50% of macaques, followed by seroconversion. In macaques inoculated intrarectally, we detected viremia, virus RNA, or both, in 100% of both species, followed by seroconversion. The magnitude and duration of infectious virus in the blood of macaques suggest humans infected with Zika virus through sexual transmission will likely generate viremias sufficient to infect competent mosquito vectors. Our results indicate that transmission of Zika virus by sexual intercourse might serve as a virus maintenance mechanism in the absence of mosquito-to-human transmission and could increase the probability of establishment and spread of Zika virus in regions where this virus is not present.

Zika virus is a member of the Spondweni serogroup, family *Flaviviridae*, genus *Flavivirus* (*1*,*2*). Since the initial isolation of the virus in 1947 (*3*), intermittent reports of Zika virus infection have been described throughout sub-Saharan Africa and Southeast Asia (*4*). Recently Zika virus extended its geographic distribution into virus-naive regions, resulting in large outbreaks in tropical regions (*5*–*7*). Most Zika virus infections are asymptomatic, and infections that are symptomatic typically cause a mild febrile illness (*2*,*5*–*7*). However, severe clinical outcomes, including congenital birth defects and Guillian-Barré syndrome, have been reported in a subset of infections (*2*,*5*–*8*).

Although the primary mechanism of Zika virus transmission is through the bite of an infective mosquito (*3*,*9*,*10*), sexual transmission involving virus strains originating from African and Asian Zika virus phylogenetic lineages has been reported (*11*–*18*). This route of transmission has been identified in nontraveling sexual partners of men who were infected with Zika virus during travel to virus-endemic regions (*11*–*15*,*17*–*19*).

Recent evidence suggests that sexual transmission of Zika virus is responsible for a substantial number of infections (*17*–*19*) and could be a virus maintenance mechanism in the absence of mosquito-to-human transmission, as well as a mechanism by which Zika virus is introduced to virus-naive regions. Viral persistence studies have demonstrated isolation of infectious Zika virus from ejaculate of a vasectomized patient 69 days postillness (*20*), detected Zika virus RNA in spermatozoa of another patient 56 days postillness (*21*), and detected Zika virus in semen specimens for >6 months after illness (*22*,*23*).

Although the titer of infectious Zika virus in semen is unknown, RNA levels of up to 7.5–8.6 log_10_ copies/mL have been reported (*13*,*21*,*24*,*25*). These data suggest that male-to-female vaginal, male-to-female anal, and male-to-male anal transmission might occur more often than previously recognized and that persons might be exposed to a higher dose of Zika virus from sexual intercourse with an infectious man than through the bite of an infective mosquito (*26*,*27*).

To model risk of Zika virus infection after sexual intercourse, we nontraumatically administered 7.0 log_10_ PFU (8.7 log_10_ copies) of the ArD 41525 Zika virus isolate into the vaginal canal or rectum of 16 adult rhesus or cynomolgus macaques and monitored them for evidence of infection through 28 days postinoculation (DPI). This dose was selected to correspond to high Zika virus RNA load(s) reported in human semen (*13*,*21*,*24*,*25*).

## Materials and Methods

### Study Design and Data Analyses

This pilot study was designed to determine if nonhuman primates (NHPs) are susceptible to Zika virus infection by the intravaginal intrarectal routes. We based sample size estimates for the 2 study groups (rhesus and cynomolgus macaques) on historic reports of experimental infections of Zika virus involving NHPs (*3*,*10*,*28*,*29*). Power analysis with a type I error rate set to 0.05 indicated that a group size of 4 animals had an 80% probability to detect Zika virus infection after intravaginal or intrarectal inoculation with the virus. This study was not designed to have the statistical power to perform analyses of chemical, hematologic, or temperature data. Investigators were not blinded during the course of the study.

### Nonhuman Primates

Research was conducted under an Institutional Animal Care and Use Committee–approved protocol at the United States Army Medical Research Institute for Infectious Diseases (Frederick, MD, USA). This protocol complied with the Animal Welfare Act, Public Health Service Policy, and other federal statutes and regulations relating to animals and experiments involving animals. The Institute is accredited by the Association for Assessment and Accreditation of Laboratory Animal Care International and adheres to principles stated in the 2011 Guide for the Care and Use of Laboratory Animals, National Research Council (https://grants.nih.gov/grants/olaw/guide-for-the-care-and-use-of-laboratory-animals.pdf).

Four female rhesus macaques from China (R1, R2, R3, and R4) and 4 female cynomolgus macaques from Cambodia (C1, C2, C3, and C4), age range 8.5–9.3 years, were individually housed during the intravaginal inoculation experiment. For the intrarectal inoculation experiment, an additional 4 rhesus macaques from China (R5, male; R6, female; R7, male; and R8, female); and 4 cynomolgus macaques from Cambodia (C5, female; C6, female; C7, male; and C8, male), age range 8.2–11.4 years, were individually housed. All macaques were prescreened and determined to be negative for Zika virus, herpes B virus, simian T-lymphotropic virus 1, simian immunodeficiency virus, simian retrovirus 1/2/3 antibodies, tuberculosis, *Salmonella* spp., *Campylobacter* spp., hypermucoviscous *Klebsiella* spp., and *Shigella* spp.

### Virus Isolate

The ArD 41525 Zika virus isolate used in this study was made from a pool of *Aedes africanus* mosquitoes collected in eastern Senegal in 1984 (passage history: AP61 cells 1, C6/36 cells 1, Vero cells 3) and has been sequenced (GenBank accession no. KU955591). We selected the ArD 41525 isolate because of its low passage history and the ancestral nature of the African phylogenetic lineage (*4*,*30*). In addition, genetic analyses of the open reading frame (ORF) of the ArD 41525 isolate from Senegal and the PRVABC59 isolate from Puerto Rico showed 88.2% nt identity and 97.3% aa identity (F. Nasar, unpub. data). Although Zika virus sequences are composed of >2 phylogenetic lineages (African and Asian), these lineages constitute a single virus serotype (*1*,*4*,*31*–*33*). Furthermore, male-to-female sexual transmission of Zika virus has involved virus strains originating from both Zika virus phylogenetic lineages (*11*–*18*). Before initiation of this study, virus challenge stocks were confirmed to be free of mycoplasma and passage-associated mutations (*4*).

### Intravaginal Virus Inoculation

For intravaginal inoculation, anesthetized macaques were placed in dorsal recumbency with their hips elevated above their torso at a 30° angle, and a 3–5-cm lubricated, size 7FR, infant feeding tube (Mallinckrodt Pharmaceuticals, St. Louis, MO, USA) was inserted into the vaginal opening. A 3-mL syringe containing 7.0 log_10_ PFU (8.7 log_10_ copies) of cell-free Zika virus suspended in 2 mL of phosphate-buffered saline (PBS) was connected to the end of the infant feeding tube and slowly administered (*34*). A 500-μL flush of 0.9% NaCl (Becton Dickinson, Franklin Lakes, NJ, USA) was then used to insure that all Zika virus inoculum was administered. Macaques stayed in dorsal recumbency with hip elevation for >20 min: R1, 26 min; R2, 23 min; R3, 21 min; R4, 20 min; C1, 21 min; C2, 30 min; C3, 28 min; and C4, 24 min.

### Intrarectal Virus Inoculation

For intrarectal inoculation, anesthetized macaques were placed in an inverted Trendelenburg position (25°–30° down angle), and a 3–5-cm lubricated, size 7FR, infant feeding tube was inserted into the rectum. A 10-mL 0.9% NaCl flush was slowly administered to soften impacted fecal material lining the rectum. After the flush, 7.0 log_10_ PFU (8.7 log_10_ copies) of cell-free Zika virus suspended in 3 mL of PBS was slowly administered (*34*), followed by a 500-μL flush of 0.9% NaCl to ensure that all Zika virus inoculum was administered. Macaques stayed in an inverted Trendelenburg position for >15 min: R5, 15 min; R6, 15 min; R7, 23 min; R8, 17 min; C5, 20 min; C6, 21 min; C7, 20 min; and C8, 20 min.

### Observations and Blood Collections

After exposure to virus, we evaluated macaques daily for signs of illness. The following clinical observations were made daily: presence or absence of rash, appearance of joints, ocular evaluation, presence or absence of blood and source, motor function, presence or absence of cough, urine output, condition of stool, and food consumption. Blood collections and physical examinations, including weight and rectal temperature, were conducted under anesthesia at −7, 1–7, 9, 12, 15, 21, and 28 DPI. Physical examinations included presence or absence of rash, capillary refill time, dehydration skin test time, joint evaluation, ocular evaluation, oral evaluation, presence or absence of blood and source, severity of bleeding if present, presence or absence of exudate and source, severity of exudate if present, presence or absence of lymphadenopathy, and lymph node size. Menstruation patterns were not recorded before inoculation. Menstruation was noted during the daily observations (0–28 DPI), but may have occurred on additional days (e.g., light or transient events).

### Chemical and Hematologic Analysis of Serum

We used comprehensive metabolic panels to test serum samples collected in 2.5-mL Z Serum Separator Clot Activator VACUETTE Tubes (Greiner Bio-One, Monroe, NC, USA) by using a Piccolo Xpress Chemistry Analyzer and Piccolo General Chemistry 13 Panel (Abbott Point of Care, Princeton, NJ, USA). Complete blood counts were performed on whole blood collected in 1.2-mL S-Monovette K3 EDTA Tubes (Sarstedt, Nümbrecht, Germany) by using a CELL-DYN 3700 system (Abbott Point of Care).

### Telemetry Devices and Monitoring

Before the study, macaques were surgically implanted with T27F-1B radio telemetry devices (Konigsberg Instruments, Pasadena, CA, USA; the telemetry unit in macaque C4 failed). The Notocord-hem Evolution Software Platform version 4.3.0.47 (Notocord Inc., Newark, NJ, USA) was used to capture and analyze data. Temperature data points were averaged and statistically filtered to remove noise and signal artifacts to generate a single data point every 30 s.

### Quantification of Infectious Virus

We performed virus titration on confluent Vero cell (CCL-81; American Type Culture Collection, Manassas, VA, USA) monolayers in 6-well plates by plaque assay. Duplicate wells were infected with 0.1-mL aliquots of serial 10-fold diluted virus in growth medium composed of Dulbecco’s modified Eagle medium (Corning Life Sciences, Tewksbury, MA, USA), supplemented with 50 μg/mL gentamicin (GIBCO, Carlsbad, CA, USA), 1.0 mmol/L sodium pyruvate, 1% vol/vol nonessential amino acids (Sigma Aldrich, St. Louis, MO, USA), and 0.4 mL of growth medium. Virus was absorbed for 1 h at 37°C and was then removed before overlaying the cell monolayers with 3 mL of 1% wt/vol Sea-Plaque agarose (Cambrex Bio Science, East Rutherford, NJ, USA) in growth medium. Cells were incubated at 37°C in an atmosphere of 5% CO_2_ for 4–5 days and then fixed with 4% formaldehyde (Fisher Scientific, Waltham, MA, USA) in PBS for 24 h. After removal of the overlay, cell monolayers were stained with 2% crystal violet (Sigma Aldrich) in 70% methanol (Sigma Aldrich) for 5–10 min at ambient temperature, and excess stain was removed with running water. Plaques were counted, and results were reported as number of PFU/mL. The lower limit of detection was 1.0 log_10_ PFU/mL.

### Extraction and Quantification of Virus RNA

To extract RNA, a serum sample (50 μL) was added to 200 μL of diethylpyrocarbonate-treated water (Ambion, Carlsbad, CA, USA), which was then added to 750 μL of TRIzol LS Reagent (Ambion). Samples were incubated for 20 min at ambient temperature. After incubation, 200 μL of chloroform (Sigma Aldrich) was added, mixed thoroughly, and incubated for 10 min at ambient temperature. After incubation, samples were centrifuged at 12,000 × *g* for 15 min at 4°C. A total of 400 μL of the aqueous phase was collected, and the RNA was precipitated by adding 1 μL of GlycoBlue (15 μg/μL) (Ambion) and 400 μL of isopropanol (Sigma Aldrich). Samples were incubated at ambient temperature for 10 min and centrifuged at 12,000 × *g* for 10 min at 4°C. The resulting pellet was then washed in 1 mL of 75% ethanol (Sigma Aldrich) and centrifuged at 12,000 × g for 5 min at 4°C, after which the pellet was air-dried for 10 min at ambient temperature and resuspended in 50 μL of diethylpyrocarbonate-treated water. Virus RNA was quantified by using a CFX96 Touch Real-Time PCR Detection System (Bio-Rad Laboratories, Hercules, CA, USA) and primers and a probe specific for the envelope gene (bases 1188–1316) (*35*). A standard curve was generated against a synthetic oligonucleotide, and genome copies were expressed as copies per milliliter. The lower limit of detection was 3.0 log_10_ copies/mL.

### Serologic Analysis

We performed plaque reduction neutralization tests (PRNTs), considered the standard for clinical diagnosis of past infection, to determine preexposure and postexposure immune responses (*36*,*37*). Serum samples were heat-inactivated at 56°C for 30 min. Samples were serially diluted 2-fold in PBS, mixed with an equal volume of 3.3 log_10_ PFU/mL of Zika virus, and incubated for 1 h at 37°C in an atmosphere of 5% CO_2_. Confluent Vero cell monolayers in 6-well plates were inoculated with 100 μL of serum/virus mixture in triplicate. Plates were incubated for 5 days at 37°C in an atmosphere of 5% CO_2_, fixed, and stained with crystal violet as described above. PRNT_80_ titers were calculated and expressed as the reciprocal of serum dilution yielding a >80% reduction in the number of plaques. Preexposure serum samples collected from rhesus macaques at −28 DPI and from cynomolgus macaques at −20 DPI showed no neutralization activity for Zika virus, indicating that these animals were not previously exposed to the virus. Postexposure serum samples were screened on 7, 15, 21 and 28 DPI.

## Results

### Intravaginally Inoculated Macaque Viremias and Antibody Responses

**Figure 1:**
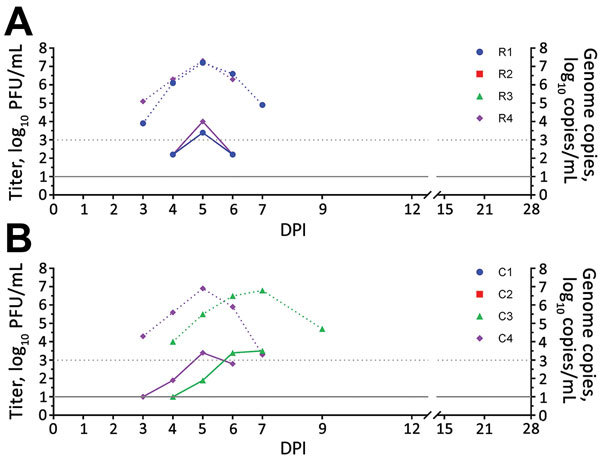
Viremia and virus RNA detected in serum of rhesus and cynomolgus macaques after intravaginal inoculation with Zika virus. A) Rhesus macaques (animals R2 and R3 showed negative results); B) cynomolgus macaques (animals C1 and C2 showed negative results). Solid lines indicate virus titers in log_10_ PFU/mL. Dotted lines indicate genome copies in log_10_ copies/mL. The lower limit of detection was 1.0 log_10_ PFU/mL for virus titers and 3.0 log_10_ copies/mL for genome copies. C, cynomolgus; DPI, days postinoculation; R, rhesus.

After intravaginal inoculation of Zika virus, 50% (2/4) of rhesus macaques and 50% (2/4) cynomolgus macaques had detectable viremias; mean peak titers were 3.8 log_10_ PFU/mL (7.2 log_10_ copies/mL) for rhesus macaques and 3.5 log_10_ PFU/mL (6.8 log_10_ copies/mL) for cynomolgus macaques (Figure 1). We detected viremia at 4–6 DPI (mean duration 3.0 d) for rhesus macaques and 3–7 DPI (mean duration 4.0 d) for cynomolgus macaques and virus RNA in serum at 3–7 DPI for rhesus macaques and 3–9 DPI for cynomolgus macaques. By 15 DPI, only those rhesus and cynomolgus macaques that showed viremia or virus RNA in serum seroconverted (R1, R4, C3, and C4), as shown by PRNT_80_ titers ranging from 1:640 to 1:1,280 (Table 1). We observed no virus neutralization for macaques R2, R3, C1, and C2 (Table 1). Menstruation was observed in all female macaques during the course of the study, but menstruation was not observed in any of the female macaques at the time of virus inoculation.

**Table 1:** Serologic responses of 8 female rhesus and cynomolgus macaques after intravaginal inoculation of Zika virus*

Macaque	Serologic response, PRNT_80_, by DPI			
	7	15	21	28
Rhesus 1	–	1:640	1:640	1:640
Rhesus 2	–	–	–	–
Rhesus 3	–	–	–	–
Rhesus 4	–	1:640	1:640	1:640
Cynomolgus 1	–	–	–	–
Cynomolgus 2	–	–	–	–
Cynomolgus 3	–	1:640	1:640	1:640
Cynomolgus 4	–	1:1,280	1:1,280	1:1,280

*Values are titers. Limit of detection was a titer of 1:20. DPI, day postinoculation; PRNT_80_, 80% plaque reduction neutralization test; –, no detectable serologic response.

### Intrarectally Inoculated Macaque Viremias and Antibody Responses

**Figure 2:**
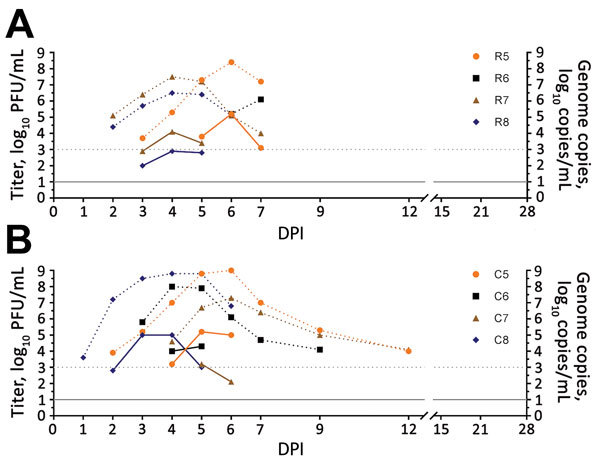
Viremia and virus RNA detected in serum of rhesus and cynomolgus macaques after intrarectal inoculation of Zika virus. A) Rhesus macaques (animal R6 showed negative results); B) cynomolgus macaques. Solid lines indicate virus titers in log_10_ PFU/mL. Dotted lines indicate genome copies in log_10_ copies/mL. The lower limit of detection was 1.0 log_10_ PFU/mL for virus titers and 3.0 log_10_ copies/mL for genome copies. C, cynomolgus; DPI, days postinoculation; R, rhesus.

After intrarectal inoculation of Zika virus, 75% (3/4) of rhesus macaques and 100% (4/4) of cynomolgus macaques had detectable viremias; mean peak titers were 4.8 log_10_ PFU/mL (8.0 log_10_ copies/mL) for rhesus macaques and 4.8 log_10_ PFU/mL (8.6 log_10_ copies/mL) for cynomolgus macaques (Figure 2). Although we did not detect viremia in 1 rhesus macaque (R6), we detected virus RNA in serum samples from this macaque at 6 DPI (5.2 log_10_ copies/mL) and 7 DPI (6.1 log_10_ copies/mL). Two cynomolgus macaques (C5, C8) had viremia levels ≥5.0 log_10_ PFU/mL for 2 days. We detected viremia at 3–7 DPI (mean duration 3.0 d) for rhesus macaques and at 2–6 DPI (mean duration 2.8 d) for cynomolgus macaques and virus RNA in serum at 2–7 DPI for rhesus macaques and 1–12 DPI for cynomolgus macaques. By 15 DPI, all rhesus and cynomolgus macaques had seroconverted (R5, R6, R7, R8, C5, C6, C7, C8), as shown by PRNT_80_ titers ranging from 1:320 to 1:1,280 (Table 2).

**Table 2:** Serologic responses of 8 rhesus and cynomolgus macaques after intrarectal inoculation of Zika virus*

Macaque	Sex	Serologic response, PRNT_80_, by DPI			
		7	15	21	28
Rhesus 5	M	–	1:1,280	1:1,280	1:1,280
Rhesus 6	F	–	1:320	1:640	1:1,280
Rhesus 7	M	–	1:1,280	1:1,280	1:1,280
Rhesus 8	F	–	1:640	1:640	1:640
Cynomolgus 5	F	–	1:640	1:640	1:1,280
Cynomolgus 6	F	–	1:640	1:640	1:1,280
Cynomolgus 7	M	–	1:640	1:1,280	1:1,280
Cynomolgus 8	M	–	1:640	1:1280	1:1280

*Values are titers. Limit of detection was a titer of 1:20. DPI, day postinoculation; PRNT_80_, 80% plaque reduction neutralization test; –, no detectable serologic response.

### Clinical Signs and Laboratory Results

We observed no overt clinical signs, including pyrexia, joint swelling, weight loss, or decreased appetite, for any of the infected macaques. The telemetry unit in macaque C6 failed during the study. Therefore, we also used rectal temperatures to determine the absence of pyrexia (Technical Appendix Figure 1). No macaque showed an increase in temperature >1.5°C from its mean rectal temperature (from readings taken at −7 and 0 DPI). Any deviations in temperature obtained by telemetry units were directly correlated with the anesthesia times or the presence of personnel in the macaque room making daily observations (e.g., physical examinations and room entry times were noted). Weights of all macaques remained relatively stable throughout the study, and no macaque showed marked weight loss (Technical Appendix Figure 2).

Potential marked increases or decreases in clinical laboratory values lasting >1 day in infected macaques were those for glucose (R1, R4, R6); blood urea nitrogen (R4, R8, C5, C6, C8); total protein (R7); alanine aminotransferase (R6, C3, C4, C5, C6, C7, C8); aspartate aminotransferase (R4, R6, C3, C4, C5, C7, C8); alkaline phosphatase (C6); total bilirubin (R8); γ-glutamyl transferase (C8); amylase (R1, R4, C3); leukocytes (R5), erythrocytes (C3, C4, C6); platelets (R1, R6, R7, R8); neutrophils (R1, R4, R6); lymphocytes (R1, R4, R6); monocytes (R1, R4, R5, R8, C3, C4, C5, C6); basophils (R1, R4, R6); and eosinophils (R1, R5, R8, C3, C4) (Technical Appendix Figures 3, 4). Macaques observed to menstruate during the study were R1 (days 4–6); R2 (days 1, 2); R3 (day 2); R4 (day 9); C1 (days 8–10); C2 (day 15); C3 (days 14, 15); and C4 (day 7).

## Discussion

Sexual transmission of Zika virus is underestimated, and its detection is confounded in regions with active mosquito-to-human virus transmission (*17*–*19*). In an effort to gauge the likelihood of infection after exposure by vaginal or anal intercourse, we inoculated the vaginal canal or rectum of rhesus and cynomolgus macaques with Zika virus. Intravaginal and intrarectal exposure resulted in infection in the absence of clinical disease, followed by seroconversion, in both species. The magnitude and duration of detectable viremia after intravaginal and intrarectal inoculation indicates that NHPs, as well as humans, could infect primary mosquito vector species.

Although the infectious dose required for primary urban and sylvatic mosquito Zika virus vectors to become infected and transmit infectious virus remains unknown, other flavivirus–vector host systems have demonstrated mosquito transmission after low-dose experimental exposure (undetectable to <3.0 log_10_ PFU/mL) (*26*,*38*,*39*). Therefore, the magnitude of viremia in some of the infected macaques was likely 10–100-fold higher than that needed to infect principal mosquito vectors. Moreover, our results suggest that sexual transmission might extend the duration of the current Zika virus epidemic and increase the probability of introduction and establishment of this virus in virus-naive regions. Likewise, sexual transmission among NHPs might be a secondary mechanism by which Zika virus is maintained in an enzootic cycle.

Despite the presence of viremia in intravaginally and intrarectally exposed macaques, overt clinical signs, such as pyrexia, rash, conjunctivitis, joint swelling, weight loss, or decreased appetite, that have been reported for some Zika virus infections in humans, were not observed in our study. Duration of viremia and clinical signs in NHPs after Zika virus infection resulting from a mosquito bite or intracranial or subcutaneous inoculation of African or Asian Zika virus isolates varies (*3*,*10*,*28*,*29*,*40*–*46*). In comparison to studies in which Zika virus was subcutaneously inoculated into NHPs (*3*,*10*,*28*,*29*,*40*–*42*), we observed a delay in detectable viremia in macaques intravaginally or intrarectally inoculated with this virus. This delay is probably the result of the virus having to infect tissues of the vaginal or rectal mucosa, replicate within these sites, and then disseminate to initiate a systemic infection. Although sentinel NHPs or those experimentally infected with Zika virus show fever or an increased temperature (*3*,*28*,*42*,*46*) or decreased appetite and weight loss (*40*), other experimental NHPs with Zika virus infection showed no overt clinical illness (*3*,*10*,*28*,*29*,*41*), which is consistent with our study results and findings for most Zika virus infections in humans (*2*,*5*–*7*).

In our study, the only clinical laboratory values that showed marked increases or decreases lasting >1 day were leukocytes, neutrophils, lymphocytes, monocytes, and basophils for rhesus macaques and alanine aminotransferase, alkaline phosphatase, γ-glutamyl transferase, amylase, erythrocytes, monocytes, and eosinophils for cynomolgus macaques. Analogous to our observations, recent studies have also reported increased levels of aspartate aminotransferase (*40*,*42*,*46*), alanine aminotransferase (*40*,*42*,*46*), and monocytes (*42*) in Zika virus–infected macaques. Although increases in some laboratory values might be the result of repeated daily anesthesia (*47*), studies with increased numbers of animals are needed to resolve which clinical laboratory parameters are associated with Zika virus infection in NHP models. Ultimately, further studies are needed to determine whether differences in the magnitude/duration of viremia and clinical signs are the result of animal genotype, virus isolate phenotype, inoculum dose, or inoculum route.

Unlike Zika virus, whose primary transmission mechanism is by mosquito bite, the primary transmission mechanism of HIV-1 is by sexual intercourse. The efficiency of HIV-1 transmission by vaginal or anal intercourse depends on a variety of factors, such as seminal viral load, number of sex acts, or co-infection (*48*,*49*). These factors also likely contribute to transmission of Zika virus. Similar to findings for other sexually transmitted viruses, such as HIV-1 (*48*), our model had a higher number of transmission events from intrarectal inoculation than intravaginal inoculation. Although the per act risk for acquiring HIV-1 infection by vaginal or anal intercourse is low (0.08%–1.7%) (*48*), risk increases proportionally with the cumulative number of sexual acts (*49*). This trend might be similar for Zika virus, for which the cumulative number of sexual acts (e.g., repeated low or moderate dose exposures) could increase risk over time.

Our experiments were conducted in a controlled research setting in which the vagina and rectum of adult macaques were nontraumatically exposed to Zika virus. Microtears induced during sexual intercourse could further enhance susceptibility to Zika virus infection in human or sylvatic NHP populations. Furthermore, preexisting sexually transmitted infections are known risk factors for increased susceptibility to secondary viral infections by vaginal or anal intercourse (*50*). Consequently, sexually transmitted infections might increase the likelihood of acquiring Zika virus through vaginal or anal intercourse.

In summary, our results indicate that sexual intercourse is a mechanism for virus transmission in the absence of mosquito-to-human transmission (i.e., effective mosquito control), as well as a mechanism by which Zika virus could be introduced to virus-naive regions and initiate human-to-mosquito transmission. Our findings highlight the need for men living in or traveling from areas to which Zika virus is endemic or epidemic to avoid unprotected sexual intercourse.

Technical Appendix Additional information on high infection rates for adult macaques after intravaginal or intrarectal inoculation with Zika virus
