# Supplementary material for: High Infection Rates for Adult Macaques after Intravaginal or Intrarectal Inoculation with Zika Virus
Source: Emerg Infect Dis. 2017 Aug;23(8):1274–81. doi: 10.3201/eid2308.170036 (PMC5547779; doi:10.3201/eid2308.170036)
Supplement: Technical Appendix — Additional information on high infection rates for adult macaques after intravaginal or intrarectal inoculation with Zika virus [file 17-0036-Techapp-s1.pdf]

Article DOI: https://doi.org/10.3201/eid2308.170036

High Infection Rates for Adult Macaques after Intravaginal or Intrarectal Inoculation with Zika Virus

Technical Appendix

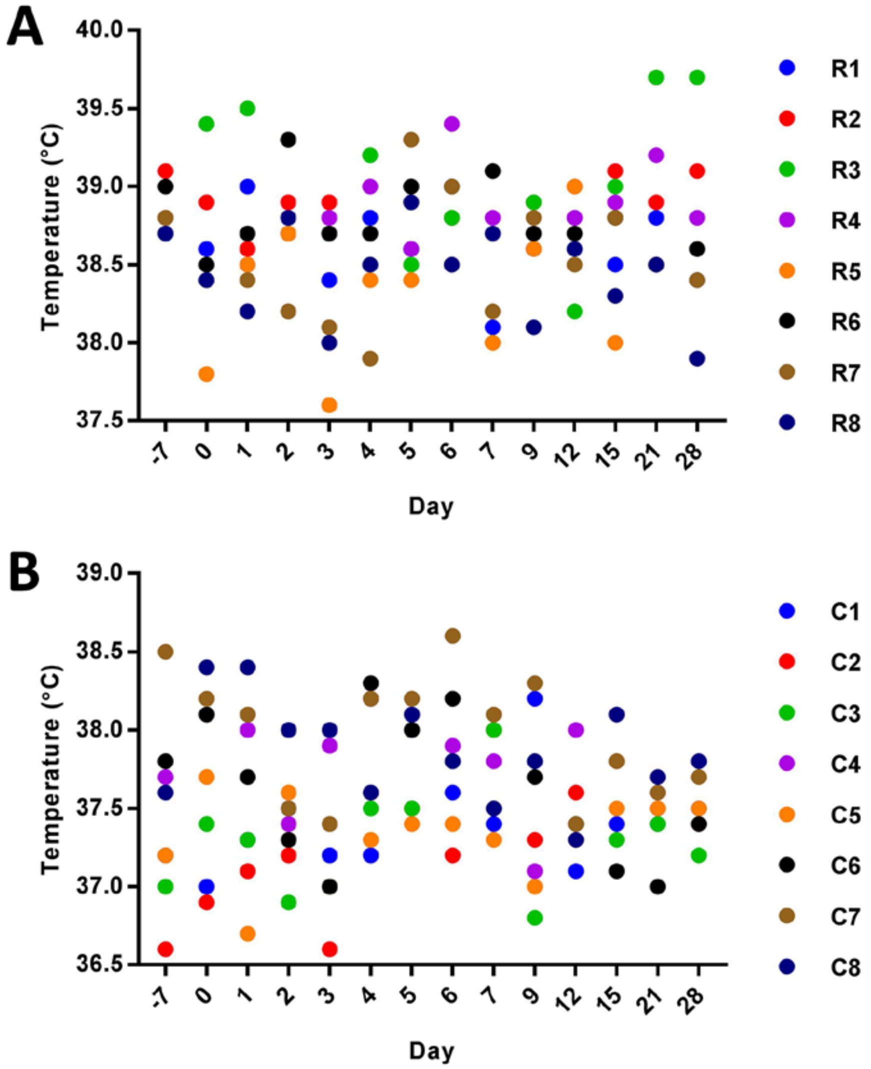

Technical Appendix Figure 1. Rectal temperatures of A) rhesus and B) cynomolgus macaques experimentally inoculated intravaginally or intrarectally with Zika virus. Day indicates day postinoculation. C, cynomolgus; R, rhesus.

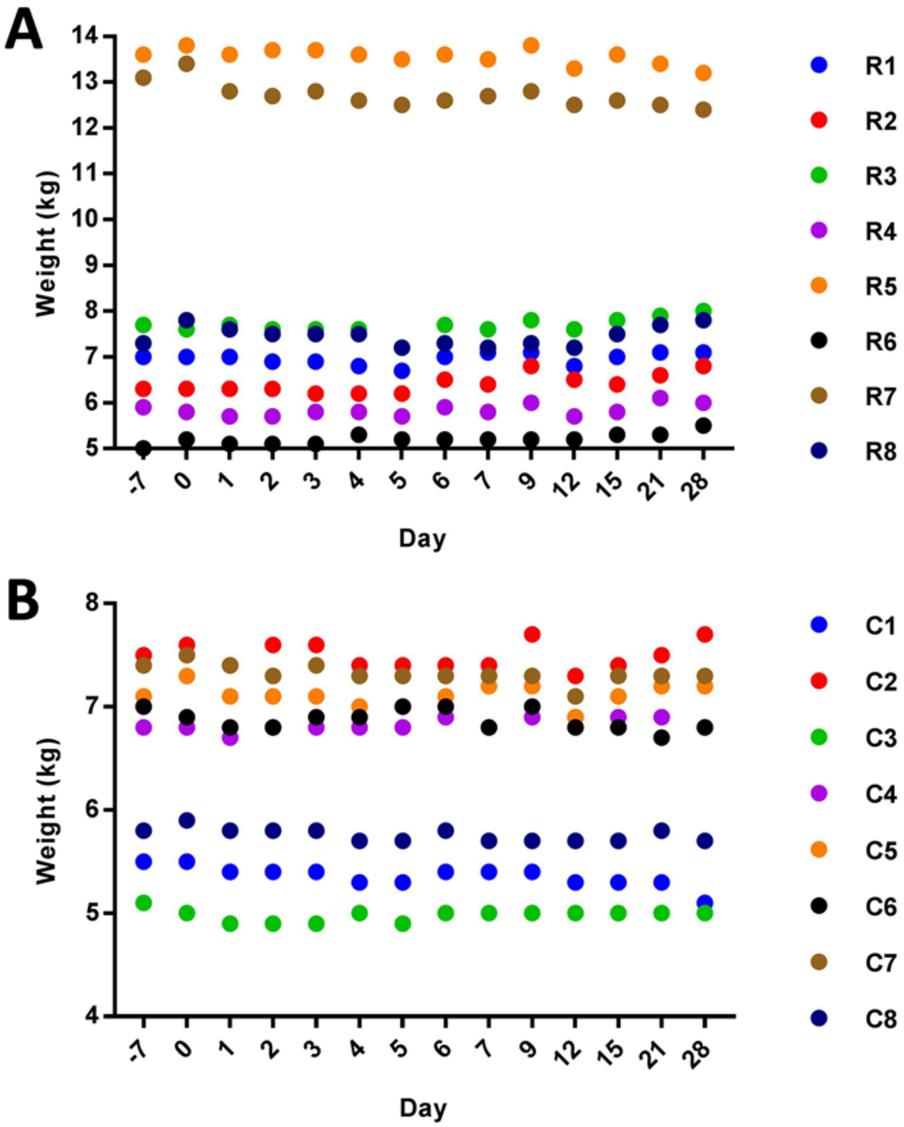

Technical Appendix Figure 2. Weights of A) rhesus and B) cynomolgus macaques experimentally inoculated intravaginally or intrarectally with Zika virus. Day indicates day postinoculation. C, cynomolgus; R, rhesus.

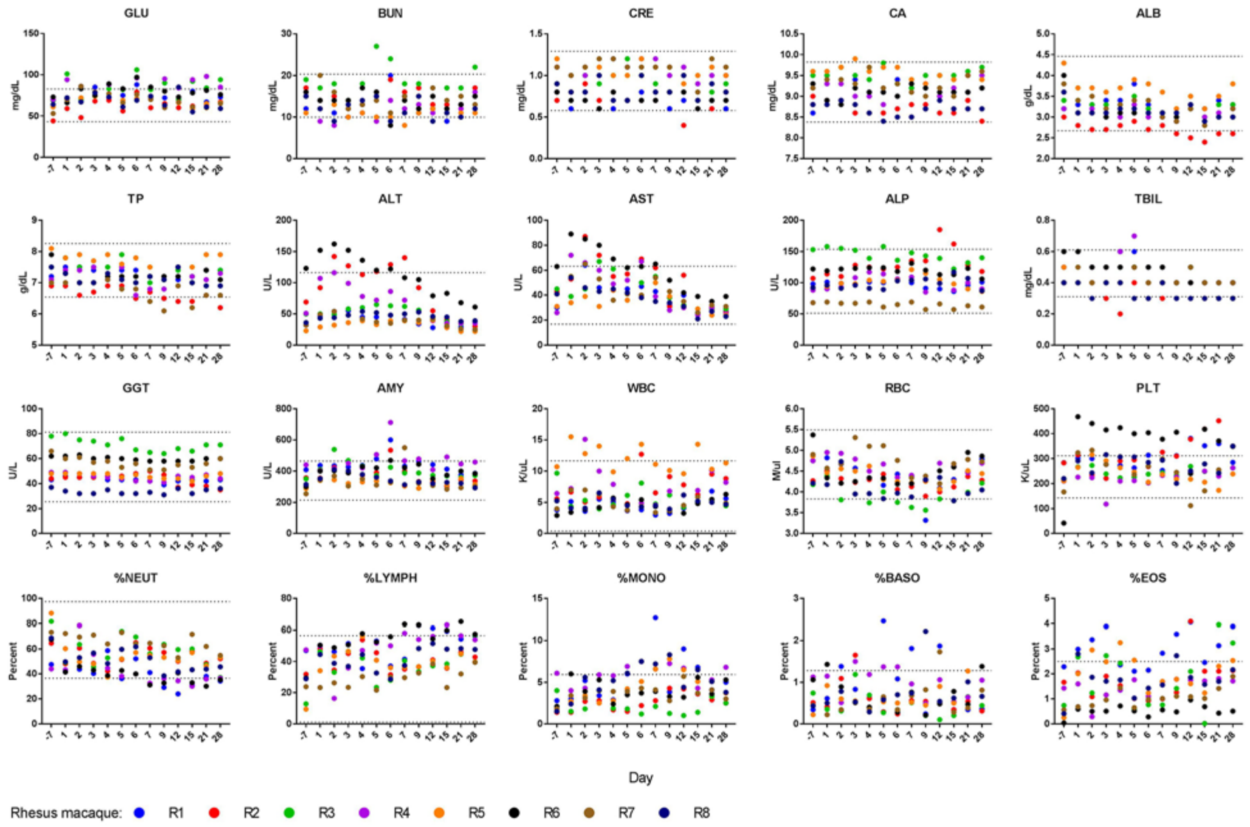

Technical Appendix Figure 3. Clinical laboratory values for rhesus macaques experimentally inoculated intravaginally or intrarectally with Zika virus. Dotted lines indicate mean ± 2 SD of corresponding laboratory value for the 8 animals at day –7. Day indicates day postinoculation. ALB, albumin; ALP, alkaline phosphatase; AMY, amylase; ALT, alanine aminotransferase; AST, aspartate aminotransferase; BASO, basophils; BUN, blood urea nitrogen; CA, calcium; CRE, C-reactive protein; EOS, eosinophils; GGT, γ-glutamyl transferase; GLU, glucose; LYMPH, lymphocytes; MONO, monocytes; NEUT, neutrophils; PLT, platelets; RBC, red blood cells; TBIL, total bilirubin; TP, total protein; leukocyte, leukocytes.

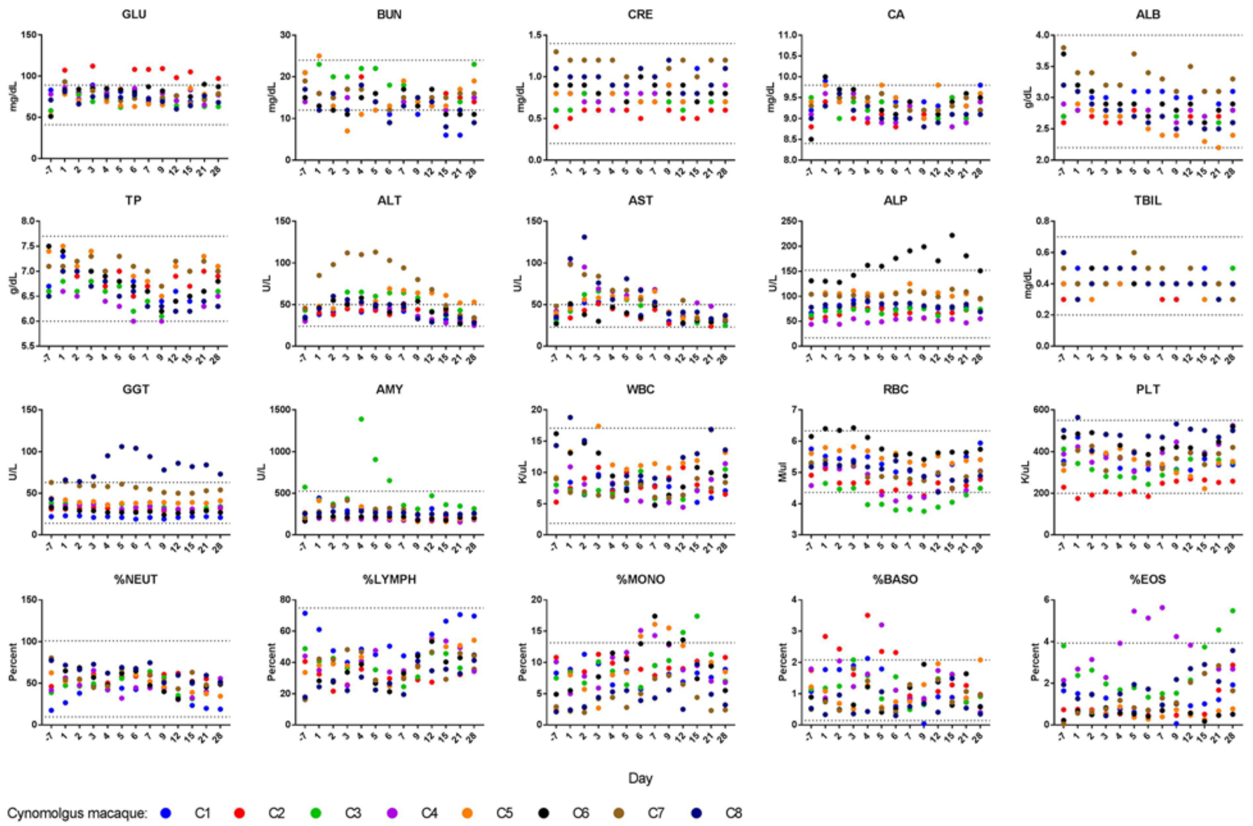

Technical Appendix Figure 4. Clinical laboratory values for cynomolgus macaques experimentally inoculated intravaginally or intrarectally with Zika virus. Dotted lines indicate mean ± 2 SD of corresponding laboratory value for the 8 animals at day –7. Day indicates day postinoculation. ALB, albumin; ALP, alkaline phosphatase; AMY, amylase; ALT, alanine aminotransferase; AST, aspartate aminotransferase; BASO, basophils; BUN, blood urea nitrogen; CA, calcium; CRE, C-reactive protein; EOS, eosinophils; GGT, γ-glutamyl transferase; GLU, glucose; LYMPH, lymphocytes; MONO, monocytes; NEUT, neutrophils; PLT, platelets; RBC, red blood cells; TBIL, total bilirubin; TP, total protein; leukocyte, leukocytes.
